# Structure of the Guanidine III Riboswitch

**DOI:** 10.1016/j.chembiol.2017.08.021

**Published:** 2017-11-16

**Authors:** Lin Huang, Jia Wang, Timothy J. Wilson, David M.J. Lilley

**Affiliations:** 1 Cancer Research UK Nucleic Acid Structure Research Group, MSI/WTB Complex, The University of Dundee, Dow Street, Dundee DD1 5EH, UK

**Keywords:** gene regulation, RNA structure, X-ray crystallography, riboregulation

## Abstract

Riboswitches are structural elements found in mRNA molecules that couple small-molecule binding to regulation of gene expression, usually by controlling transcription or translation. We have determined high-resolution crystal structures of the *ykkC* guanidine III riboswitch from *Thermobifida fusca*. The riboswitch forms a classic H-type pseudoknot that includes a triple helix that is continuous with a central core of conserved nucleotides. These form a left-handed helical ramp of inter-nucleotide interactions, generating the guanidinium cation binding site. The ligand is hydrogen bonded to the Hoogsteen edges of two guanine bases. The binding pocket has a side opening that can accommodate a small side chain, shown by structures with bound methylguanidine, aminoguanidine, ethylguanidine, and agmatine. Comparison of the new structure with those of the guanidine I and II riboswitches reveals that evolution generated three different structural solutions for guanidine binding and subsequent gene regulation, although with some common elements.

## Highlights

• The guanidine III riboswitch forms a pseudoknot with a triplex section
• The conserved core forms a left-handed helical ramp that binds a guanidine molecule
• Guanidine is H-bonded to two guanine bases, a 2′-OH, and makes a π-cation interaction
• Four modified guanidine compounds also bind to the pocket, which is open on one side

## Graphical Abstract

**Figure:**
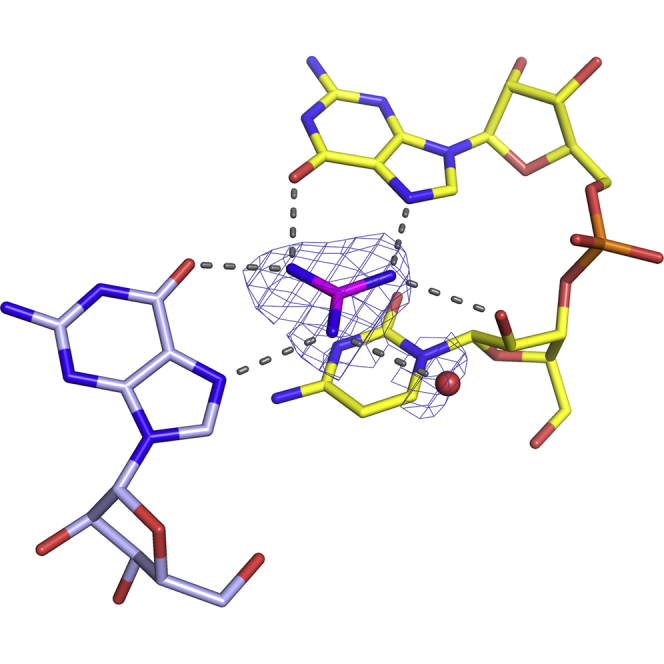

## Teaser

Huang et al. show that the guanidine III riboswitch folds as a pseudoknot that includes a triple helix. The triplex is continuous with the core that forms a left-handed helical ramp that generates the guanidine binding site. Guanidine is hydrogen bonded to the Hoogsteen edge of two guanine bases, and a 2′-hydroxyl. Guanidine binding stabilizes the triplex, leading to gene activation.

## Introduction

Riboswitches are bacterial *cis*-acting genetic regulatory elements that reside in mRNA molecules (Roth and Breaker, 2009, Serganov and Nudler, 2013). They function by binding small molecules so as to alter the local conformation, thereby switching on or off gene expression either at the transcriptional or translational level in most cases. The binding ligand will be related in some manner to the metabolic role of the gene product, such as an enzyme or transporter. To function effectively it is important that the ligand binding is highly selective, and discriminates against metabolites of similar chemical structure. In some cases multiple different riboswitches have evolved to respond to the same ligand, exemplified by riboswitches for S-adenosyl methionine (five classes known) (Corbino et al., 2005, Fuchs et al., 2006, Poiata et al., 2009, Weinberg et al., 2008, Winkler et al., 2003) and preQ1 (two classes) (Meyer et al., 2008, Roth et al., 2007).

**Figure 1:**
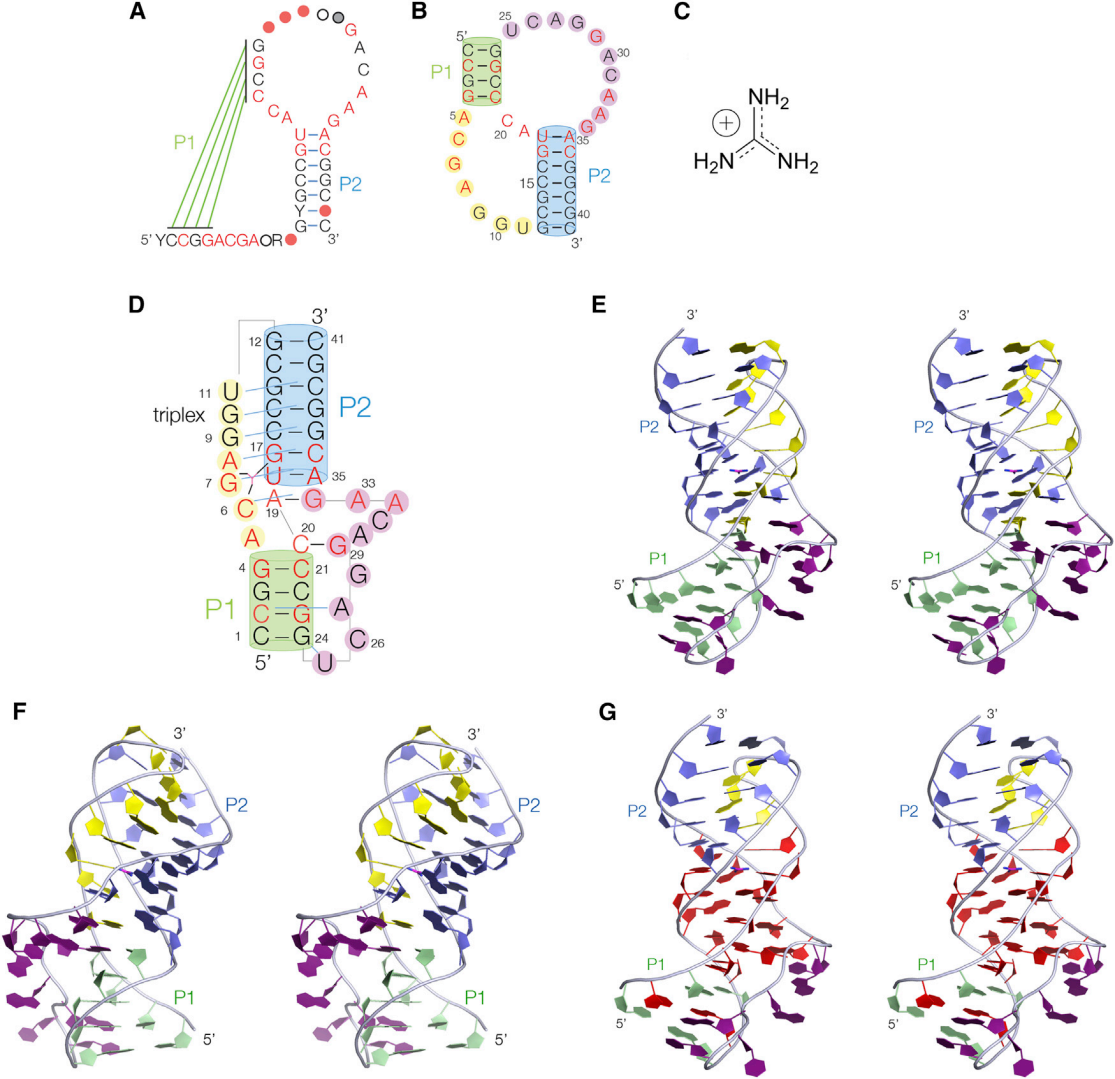
The Overall Conformation of the Guanidine III Riboswitch Parallel-eye stereoscopic pairs are shown for the molecular graphics images. (A) Guanidine III riboswitch sequence and secondary structure (adapted from Sherlock et al., 2017). Nucleotides colored red are >97% identical in all species analyzed. Nucleotides are present at the positions shown by circles with 97% (red), 90% (black), 75% (gray), and 50% (open) probability. (B) The *Thermobifida fusca ykkC* guanidine III riboswitch sequence drawn in the pseudoknot secondary structure, comprising stem-loops P1 and P2. (C) The structure of the guanidinium cation. (D) Schematic illustration of the structure. Nucleotides colored red are >97% identical in all species analyzed. Tertiary hydrogen bonding interactions are indicated by cyan-colored lines. The color scheme shown here is used throughout Figures 2, 3, 4, 5 and 6. (E and F) Two views of the crystal structure, from opposite sides. (G) View of the crystal structure, from the same perspective as (E), in which nucleotides that are conserved with >97% identity are colored red. Note that the sequence of the entire central section of the riboswitch is conserved.

The *ykkC* family of structural motifs were identified as probable riboswitches more than a decade ago (Barrick et al., 2004), but their assignment to a specific ligand occurred only recently. It was found that they respond to guanidine (Figure 1) (Nelson et al., 2017), which was not fully appreciated to be a metabolite in bacterial cells. In fact this compound is highly toxic, and cells must be detoxified by proteins that either convert it to less harmful species (e.g., carboxylases), or transport it out of the cell (efflux pumps). Elevated concentrations of guanidine require that expression of these gene products is induced, and therefore the riboswitches function as ON switches. Because of its high p*K*_a_ (13.6; Perrin, 1966), guanidine will be protonated at physiological pH, existing as the guanidinium cation with six protons and D_3h_ symmetry. However we shall in general refer to this as guanidine except where its charge is relevant.

Breaker and colleagues have shown that three classes of these riboswitches exist, now termed the guanidine I (Nelson et al., 2017), II (Sherlock et al., 2017), and III (Sherlock and Breaker, 2017) riboswitches. The guanidine I riboswitch was shown to operate at the transcriptional level (Nelson et al., 2017), while the proximity of the guanidine II and III riboswitches to ribosome binding sites suggested that these would act at the translational level (Sherlock and Breaker, 2017, Sherlock et al., 2017). Each riboswitch responds to guanidine in the micromolar concentration range, yet their predicted secondary structures suggested quite different RNA folds for the three classes. Crystal structures for the guanidine I (Battaglia et al., 2017, Reiss et al., 2017) and II riboswitches (Huang et al., 2017, Reiss and Strobel, 2017) have been solved recently. The guanidine I riboswitch folds by side-by-side packing of two long stem-loops, creating a guanidine binding site with components from both (Battaglia et al., 2017, Reiss et al., 2017). By comparison, the guanidine II riboswitch is generated by loop-loop interaction between two closely similar, relatively short stem-loop structures, creating binding sites for two guanidine molecules at the interface (Huang et al., 2017, Reiss and Strobel, 2017). While not identical the binding sites of the two riboswitches contain a number of similar features, both being rich in guanine nucleobases. The guanidine ligands are extensively hydrogen bonded to the RNA in the binding site, notably to the O6 and N7 atoms on the Hoogsteen edge of a guanine. In addition the positively charged guanidinium ions are stacked on another guanine nucleobase making a π-cation interaction (Gallivan and Dougherty, 1999, Wintjens et al., 2000).

The sequence and predicted secondary structure of the guanidine III riboswitch suggests that it folds as a standard pseudoknot structure (Sherlock and Breaker, 2017) comprising two interconnected stem-loop structures P1 and P2 (Figure 1). The structure is therefore unlikely to be related to either of the other two classes. Using in-line probing data Sherlock et al. (2017) showed that guanidine bound to the RNA with an affinity of 60 μm and a Hill coefficient of close to unity, consistent with the binding of a single molecule of the ligand. Moreover they showed that the guanidine III riboswitch has a slightly lower specificity compared with the guanidine I riboswitch, binding methylguanidine and some other small variants (Sherlock and Breaker, 2017). This was also the case for the guanidine II riboswitch (Sherlock et al., 2017), where it was shown that these ligands could be accommodated because of a small side opening to the binding pocket (Huang et al., 2017).

In this work we have crystallized the guanidine III riboswitch and solved its structure by X-ray crystallography at a resolution of 1.91 Å. We find that the RNA adopts a completely different fold from the other two riboswitches, being based on a classic H-type pseudoknot (Rietveld et al., 1982) structure. This includes a triple helical section that generates the guanidine binding site and is thus stabilized by guanidine binding. The availability of three different guanidine riboswitch structures allows us to make interesting comparisons, revealing both common themes and interesting differences that illustrate how RNA is a versatile and remarkable macromolecule for binding small molecules.

## Results

### Construction and Crystallography

A 41 nt RNA corresponding to the *Thermobifida fusca ykkC* guanidine III riboswitch (Figure 1B) was made by chemical synthesis. This included two 5-bromocytosine nucleotides used to provide phase information by SAD. RNA was crystallized with or without added ligands (Table S1). The majority of species crystallized in the trigonal space group P3_1_21. Crystals of the complex with guanidine were obtained in P3_1_21 at 1.91 Å resolution (PDB: 5NWQ), but were also obtained in space group P3_2_12 at 2.94 Å resolution (PDB: 5NZ6). The conformations were essentially identical in both crystal forms (Figure S1), with an all-atom root-mean-square deviation (RMSD) = 0.55 Å. Details of data collection and refinement statistics for all the crystallographic data as deposited with the PDB are shown in Table S2.

### Overall Structure

The single continuous strand of the guanidine III riboswitch RNA adopts the structure of an H-type pseudoknot motif, based upon two Watson-Crick-paired stem-loops P1 and P2, in which part of both terminal loops base pair to form the other stem in each case (Figures 1 and S2). Running from the 5′ terminus (placed at the lower end of the depiction in Figures 1E and 1F), the RNA initially forms the ascending strand of P1, then continuing as the third strand in the major groove of P2. It then makes a sharp turn before forming the descending strand of P2, passing through the core, and then forming the descending strand of P1. It then turns before running along the minor groove of P1, passing through the core and finally forming the ascending strand of P2 before reaching its 3′ terminus. The non-Watson-Crick paired strands make a large number of hydrogen bonding contacts predominantly with the nucleobases that will be discussed in more detail, and these are tabulated in Table S3. All the inter-nucleotide interactions are plotted in Figure S3, graphically revealing P1 and P2 as sequential Watson-Crick base pairs (red) and the numerous additional interactions. The central core region of the riboswitch adopts an extensively hydrogen-bonded structure that includes the guanidine binding site as we discuss below.

### The P1 Helix

**Figure 2:**
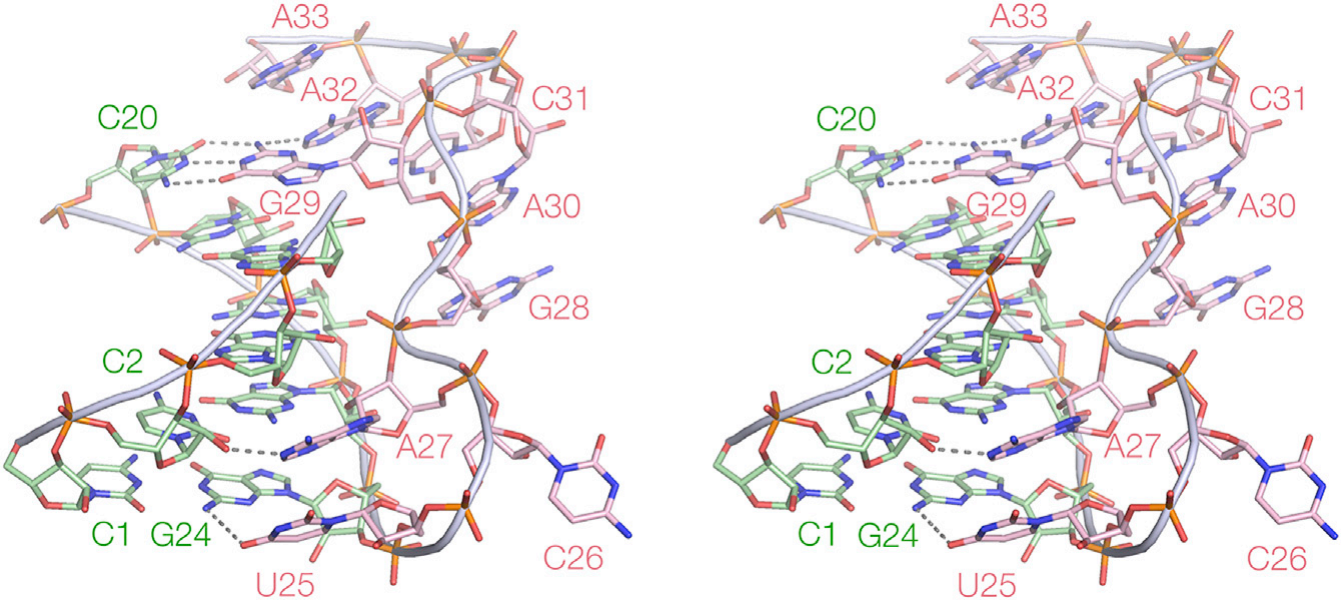
A Parallel-Eye Stereoscopic View of the P1 Helix Plus the Extra Strand Key long-range hydrogen bonds are shown by broken lines.

The P1 helix begins with the first four nucleotides (1–4) base pairing with nucleotides 21–24 (Figure 2). A sharp turn in the backbone follows G24, and U25 and A27 make hydrogen bonding interactions in the minor groove of P1. G29 is inserted into the helix and is stacked on G4, making a Watson-Crick base pair with C20, which is the fifth base pair of P1. P1 ends with a base pair between A5 and A19 (*trans* Watson-Crick:Hoogsteen). There are relatively few contacts between the third stand and nucleotides of the P1 helix, but instead G28, A30, C31, A32, and A33 form a continuous stack of nucleobases before passing into P2.

### The P2 Triple Helix

**Figure 3:**
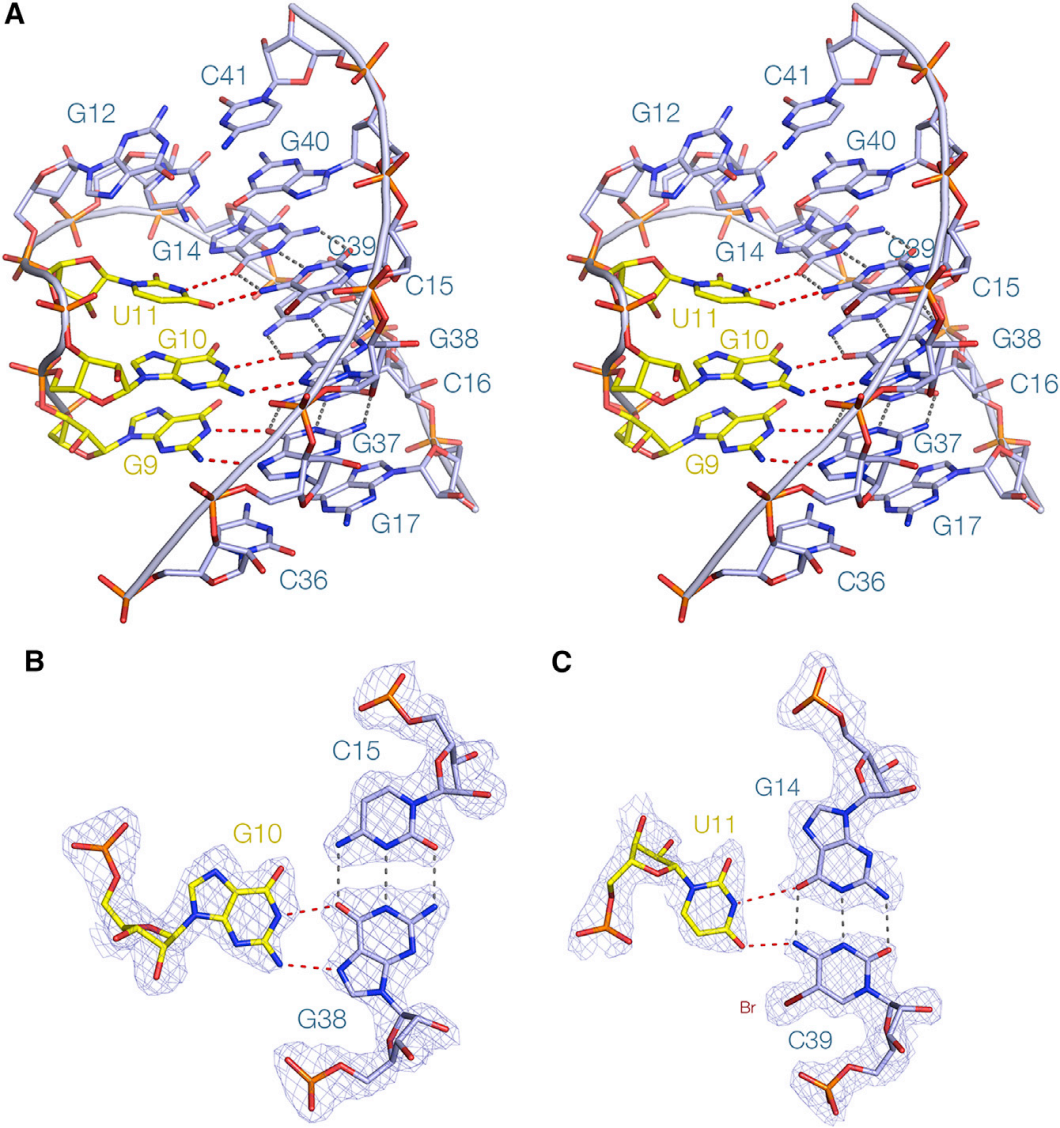
A Section of the P2 Regular Triple Helix and Two Representative Nucleotide Triplets Note, the regular triple helical section is continuous with the helical ramp shown in Figure 4. (A) A parallel-eye stereoscopic representation of a section of the triplex region. Hydrogen bonds between the third strand (yellow) and the P2 base pairs are highlighted in red. A view down the axis of the triple helix is presented in Figure S4. (B and C) The structures of the nucleotide triplets involving G10 and U11. Sections of the experimental phasing map contoured at 1.2 σ are shown. C39 is 5-bromocytosine (Br) used to phase the diffraction.

P2 comprises Watson-Crick base pairs between nucleotides 12–18 and 35–41. While the first two are simple base pairs, the remaining five base pairs all form interactions with the third strand (C6–U11) in the major groove, so that much of P2 is really a triple helix. The section that includes G9, G10, and U11 is a regular triple helix with discrete base triples (Figures 3 and S3). Below this the triple helix is less regular, forming the helical ramp discussed in the following section. This includes the guanidine binding site. The third strand of P2 is the continuation of the strand of P1 running from the free 5′ terminus, and thus the base-base interactions with the P2 nucleotides G34–C39 can be considered an extension of P1. This can be observed in the 2D interactions plot (Figure S3), running at 90° to the P2 interactions. In contrast to the third strand of the P1 helix, every nucleotide of the third strand of P2 makes hydrogen bonds in the major groove (see Table S3).

### The Conserved Central Core and the Helical Ramp

**Figure 4:**
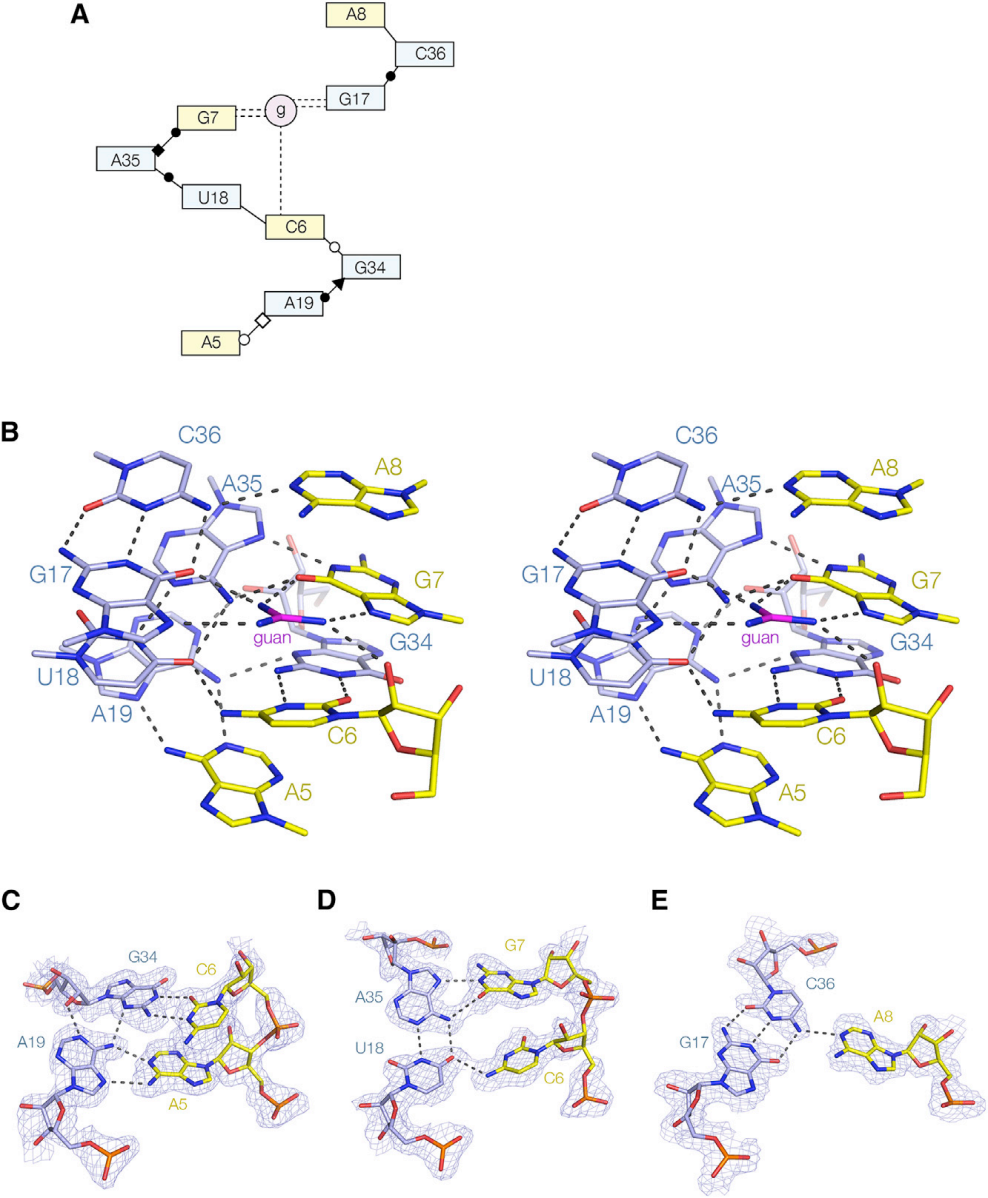
The Helical Ramp (A) Scheme of the chain of interactions between ten nucleotides and the guanidine ligand. The Leontis and Westhof (2001) nomenclature has been used to classify the interactions where possible. Broken lines denote hydrogen bonds to the guanidine ligand (g). (B) A parallel eye stereoscopic view of the helical ramp, from A5 to A8. The continuous set of inter-nucleotide interactions form a gentle left-handed helix. In the interest of clarity the ribose-phosphate backbone is not displayed except where the ribose makes a direct interaction. (C–E) Three sections of the ramp shown in greater detail together with their experimental phasing maps contoured at 1.2 σ. Note that the second and third sections depicted are connected via the guanidine bound to G7 and G17 (see Figure 5).

Highlighting the nucleotides in the structure of the riboswitch by their degree of conservation (Figure 1G) reveals that every nucleotide in the core that lies between the P1 and P2 helical domains is >97% conserved. The core comprises the lower part of the P2 triple helix and the interface with P1. Within this region there is a continuous chain of interactions connecting ten conserved nucleobases plus the guanidine ligand. All involve at least one hydrogen bond, and in six cases two hydrogen bonds. The connected nucleobases adopt a gentle left-handed helical ramp (Figure 4); the chain of connectivities is also shown in the lower half of Figure S3. The final member of this chain of nucleobases is A8, but above this (but not hydrogen bonded to it) is the triplet formed from C16–G37⋅G9, where G9 is contributed by the third stand in the major groove of the C16–G37 base pair. Two further triplets lie on top of this, i.e., C15–G38⋅G10 and G14–C39⋅U11 (see Figure 3).

### The Guanidine Binding Site

**Figure 5:**
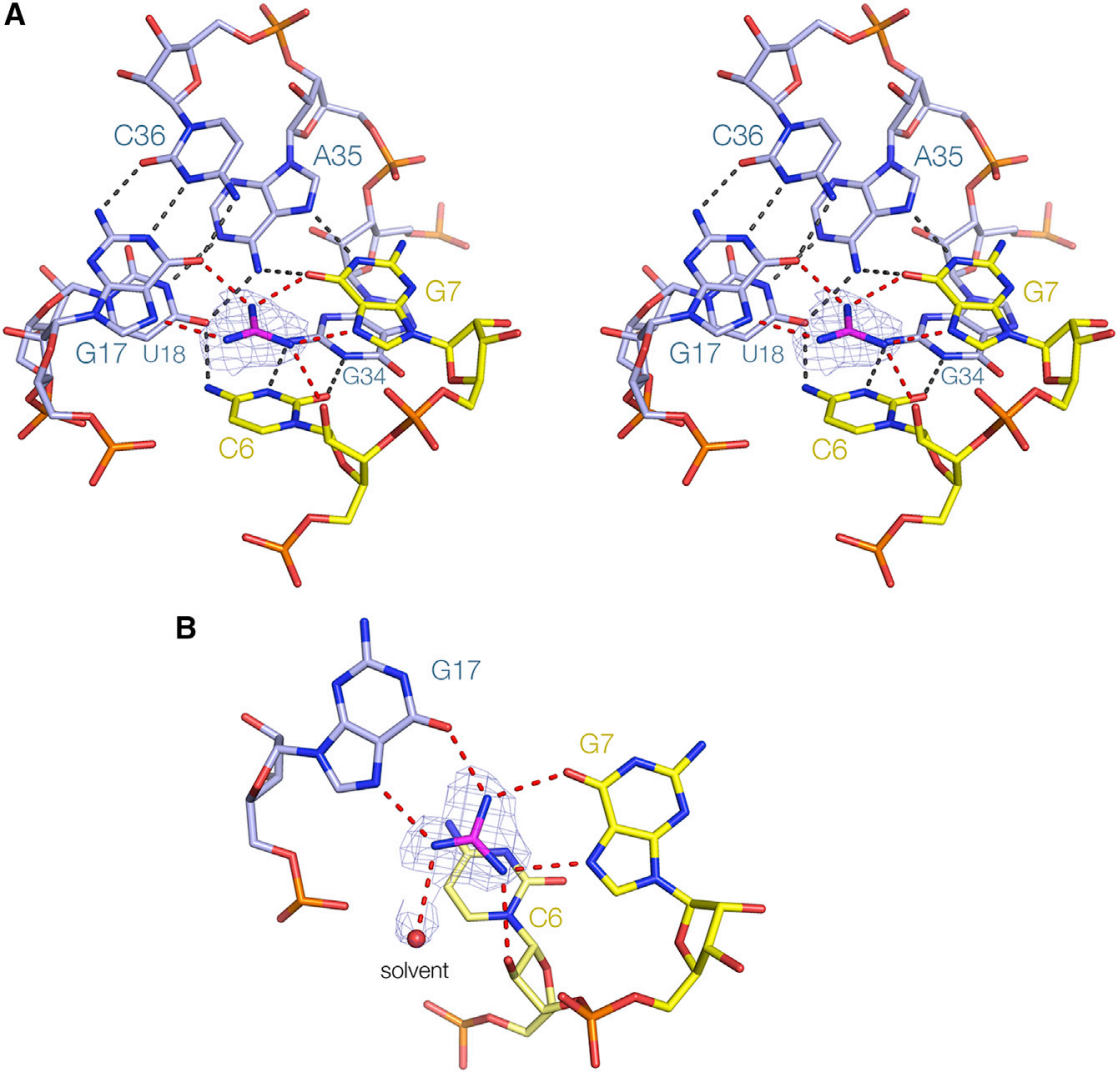
The Guanidine Binding Site of the Guanidine III Riboswitch The experimental phasing maps contoured at 1.2 σ is shown for the guanidine ligand. The guanidine is colored magenta. Hydrogen bonds donated by guanidinium protons are colored red. Note that the guanidinium cation is stacked upon the nucleobase of C6, making a π-cation interaction. (A) A parallel-eye stereoscopic view of the binding pocket. (B) A view down onto the guanidine, simplified to show only nucleotides involved in direct interaction with the ligand. The electron density for a solvent molecule (presumed water) has been included in this image.

The helical ramp creates the guanidine binding site of the riboswitch (Figure 5). Two of the guanidino nitrogen atoms are hydrogen bonded to O6 and N7 on the Hoogsteen edge of G7, while two are equivalently bonded to G17, so that one ligand nitrogen is hydrogen bonded to the O6 atoms of both G7 and G17 nucleobases. Hydrogen bonding between guanidine and the Hoogsteen edge of guanine also occurs in guanidine riboswitches I (Battaglia et al., 2017, Reiss et al., 2017) and II (Huang et al., 2017), but this is the first case where the ligand is simultaneously bonded to two guanine nucleobases in this same manner. The nitrogen atom that is bonded to N7 of G7 is also hydrogen bonded to the O2′ of the ribose of C6, but the nitrogen atom bonded to N7 of G17 only makes the one interaction with the RNA. Thus while five of the six guanidino protons are hydrogen bonded to RNA ligands, the sixth is not. However the experimental phasing map (Figure 5B) shows clear density in the position that would be occupied by a sixth acceptor, which we tentatively assign as a water molecule. Lastly, we observe that the guanidine is stacked on the nucleobase of C6 at a distance of 3.5 Å. This is a π-cation interaction (Gallivan and Dougherty, 1999, Wintjens et al., 2000) between the positively charged guanidino cation and the aromatic nucleobase. Similar π-cation interactions are also observed in guanidine riboswitches I and II (Battaglia et al., 2017, Huang et al., 2017, Reiss and Strobel, 2017, Reiss et al., 2017), except that the participating nucleobases are guanine in those structures.

### A Degree of Flexibility in Ligand Binding

**Figure 6:**
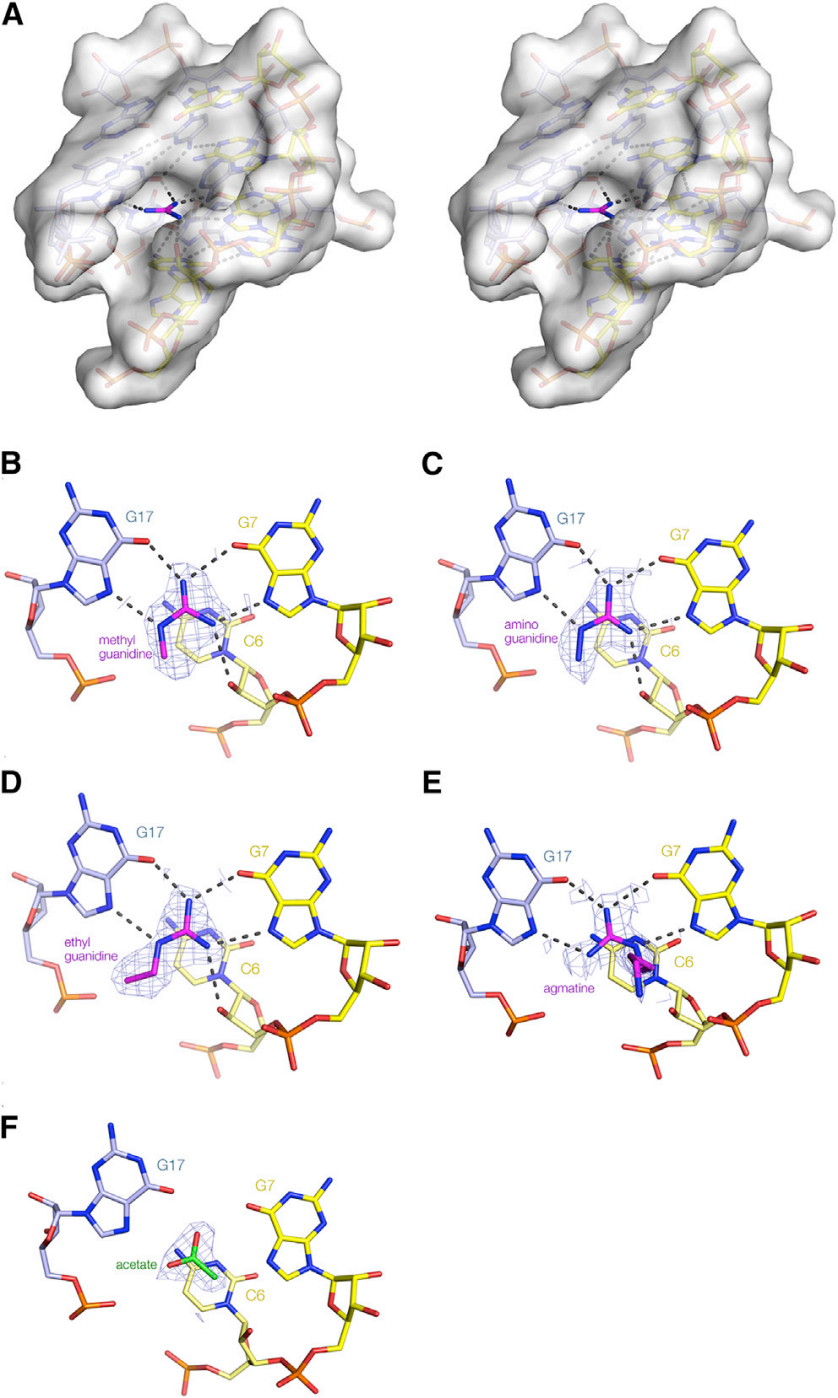
Binding of Guanidine Variants of the Guanidine III Riboswitch (A) View into binding pocket showing the side opening. A parallel-eye stereoscopic pair is shown. A transparent surface has been displayed on the riboswitch structure. The guanidine ligand is shown in stick form, colored magenta, and is clearly visible in the cleft in the RNA. (B–F) Alternative ligands bound into the binding pocket of the riboswitch. The experimental phasing maps contoured at 1.2 σ are shown for the ligands. The guanidine variants are colored magenta, and acetate colored green. Ligands: (B) methylguanidine, (C) aminoguanidine, (D) ethylguanidine, (E) agmatine, and (F) acetate bound into the riboswitch crystallized in the absence of added ligand. Further views of the agmatine complex are shown in Figure S5.

The guanidine group, the nucleobases of G7 and G17 and the O2′ of C6 are all approximately co-planar. Altogether five of the six protons of the guanidinium cation are involved in hydrogen bonding interactions to macromolecular ligands, and all have excellent geometry. However the remaining proton of the nitrogen atom that is bonded to N7 of G17 is not involved in hydrogen bonding to RNA, but rather to solvent. Examination of a space-filling representation of the guanidine III riboswitch structure shows that there is an opening in the side facing the proton that is unbonded to RNA (Figure 6A). Using in-line probing experiments, Sherlock and Breaker (2017) showed that methylguanidine and aminoguanidine bound to the riboswitch with only a small loss of affinity, suggesting that the additional methyl and amino groups could be accommodated in this space. Ethylguanidine and agmatine (i.e., guanidine with a butylamine side chain) were also found to bind the guanidine III riboswitch, with reduced affinities (Sherlock and Breaker, 2017).

Methylguanidine and aminoguanidine were co-crystallized with the riboswitch RNA, while ethylguanidine and agmatine were soaked into ligand-free crystals. All crystallized in the trigonal space group P3_1_21, and all diffracted to good resolution (2.01–2.32 Å; Table S2). The structures were solved by SAD using the bromine atoms. Methylguanidine, aminoguanidine, and ethylguanidine were each bound in the same way as guanidine (Figures 6B–6D). Each nitrogen atom was hydrogen bonded to G7, G17, and C6 in the same manner as unmodified guanidine (Figure 5), with the additional methyl, amino, or ethyl groups attached to the nitrogen hydrogen bonded to G17 N7. The appended side chains were co-planar with the guanidine atoms, and directed laterally through the side opening. However, the side opening cannot readily accommodate longer alkyl chains, and agmatine was bound in a significantly different manner (Figures 6E and S5). The ligand was found to be rotated by 120° in the binding pocket so that the butylamine side chain is attached to the guanidine nitrogen that is hydrogen bonded to G7 N7, and consequently there is no hydrogen bond to C6 O2'. The trajectory of the butylamine side chain is quite different from those of the other ligands, being directed upwards along the groove in the RNA structure (Figure S5B).

### The Structure of the Binding Site in the Absence of Guanidine Derivatives

We also solved the structure of the riboswitch in the absence of added ligand, which crystallized in multiple space groups; the structure was determined in P2_1_2_1_2_1_. In the crystal, the structure of the riboswitch is closely similar to that of the bound forms (RMSD = 0.54 Å), with the binding pocket adopting the same conformation. The RNA was crystallized in the presence of 0.1 M acetate, and we found that one molecule of acetate (or most probably acetic acid, given that the crystals were formed at pH 5.6) had bound in the ligand binding pocket, stacked onto the nucleobase of C6 in a closely similar manner to guanidine (Figure 6F). We have chosen a rotational setting that is most consistent with the apparent bond lengths and which places the methyl group over the C6 nucleobase. However, we cannot rule out an alternative rotational setting, or even a degree of static disorder in this respect.

## Discussion

The guanidine III riboswitch adopts a classic H-type pseudoknot structure containing a remarkable novel helical ramp structure. The P2 helix forms a regular triple helical structure by interaction with the third strand in the major groove, which then passes into a more irregular region (i.e., the helical ramp) in which all the component nucleotides are strongly conserved. This highly unusual structure generates the guanidine ligand binding site.

The ramp comprises a chain of ten nucleobases contributed by both strands of P2 and the third strand, and includes guanidine as an integral, hydrogen-bonded component. This is immediately continuous with the three nucleotide triples of the regular triplex. The binding of the guanidine ligand should thus stabilize the ramp and triplex structure. In-line probing data of Sherlock et al. (2017) show that while the P1 and P2 stems remain uncleaved in the absence of guanidine, i.e., adopting the duplex conformation under all conditions, the third strand (the section comprising A5 to G12 in our structure, colored yellow) is strongly cleaved in the absence of ligand, but exhibits a marked reduction in the extent of cleavage on addition of guanidine. Under physiological conditions it is therefore likely that this strand is disengaged from the P2 duplex in the absence of ligand, moving into the helix to form the triplex and the helical ramp on binding guanidine. Triple-base interactions have been noted in other pseudoknot structures (Theimer et al., 2005), and in single-molecule force-extension experiments Tinoco and coworkers (Chen et al., 2009) demonstrated that formation of nucleotide triplets enhance the mechanical stability of H-type pseudoknot structures, supporting this view.

The 3′ end of the *T. fusca ykkC* guanidine III riboswitch is 16 nt from a ribosome binding site, and the whole region has the propensity to undergo secondary structure formation. It is therefore probable that binding guanidine stabilizes the fully folded form of the riboswitch, including the ramp and adjacent regular triplex structure thereby destabilizing competing structures and thus exposing the ribosome binding site so that translation of the mRNA can be initiated. This switches on the gene to make a protein required to counter the toxicity of guanidine in the cell.

One of the important properties required of the guanidine riboswitches is to distinguish the correct ligand from the rather similar urea molecule. The binding site of the guanidine III riboswitch shows how this is achieved. All three guanidino nitrogen atoms are hydrogen bonded to guanine O6 and N7 atoms that can only accept hydrogen bonds. In principle C6 O2′ could donate a hydrogen bond to a urea oxygen atom, but the C2′-endo sugar pucker enables C6 O2′ to instead donate a hydrogen bond to the *pro*R oxygen of the phosphate of G7. Urea is unprotonated at physiological pH so the oxygen has no proton to donate. Furthermore, binding of neutral urea will not be stabilized by the π-cation interaction. We found that acetate (or more probably acetic acid) bound in the ligand binding site when crystals formed in the absence of added guanidine derivatives. Under these conditions we would expect the site to be occupied with components from the solvent, having previously observed ammonium ions in the binding site of the guanidine II riboswitch (Huang et al., 2017). Since acetate was present at 0.1 M during crystal formation this has clearly entered the binding site, but is unlikely to contribute significantly to the stabilization of the structure.

We may also ask why arginine is not a ligand for the guanidine III riboswitch. The side opening can accommodate a range of small side chains, with affinities in the order guanidine > methylguanidine ∼ aminoguanidine > ethylguanidine > agmatine (Sherlock and Breaker, 2017). In principle arginine might be accommodated in the same manner as agmatine, but it is likely that the additional carboxylate group of the amino acid adds further destabilization leading to an unmeasurable affinity (Sherlock and Breaker, 2017).

Driven by the imperative of ridding the cell of the toxic effects of guanidine in a controlled manner, evolution has found three different solutions to the problem of folding RNA into structures that bind this compound with high specificity and micromolar affinity (Table S4). The availability of high-resolution crystal structures for three quite different riboswitches that have evolved to bind the same guanidine ligand now provides an opportunity to make some interesting comparisons.

We have found that the guanidine III riboswitch adopts a totally different fold from the guanidine I and II riboswitches (Battaglia et al., 2017, Huang et al., 2017, Reiss and Strobel, 2017, Reiss et al., 2017). The overall fold of this riboswitch is that of a pseudoknot, leading to the formation of a triple helix (the P2 helix) that sets up the guanidine binding site. By comparison, the guanidine I riboswitch comprises two stem-loops (one of which is mutually incompatible with a transcriptional terminator) that pack side-by-side to create the guanidine binding site (Battaglia et al., 2017, Reiss et al., 2017). The guanidine II riboswitch also involves two stem-loops, but these associate by intimate loop-loop interactions that create two guanidine binding sites (Huang et al., 2017, Reiss and Strobel, 2017), leading to cooperative binding of the ligand in this riboswitch.

**Figure 7:**
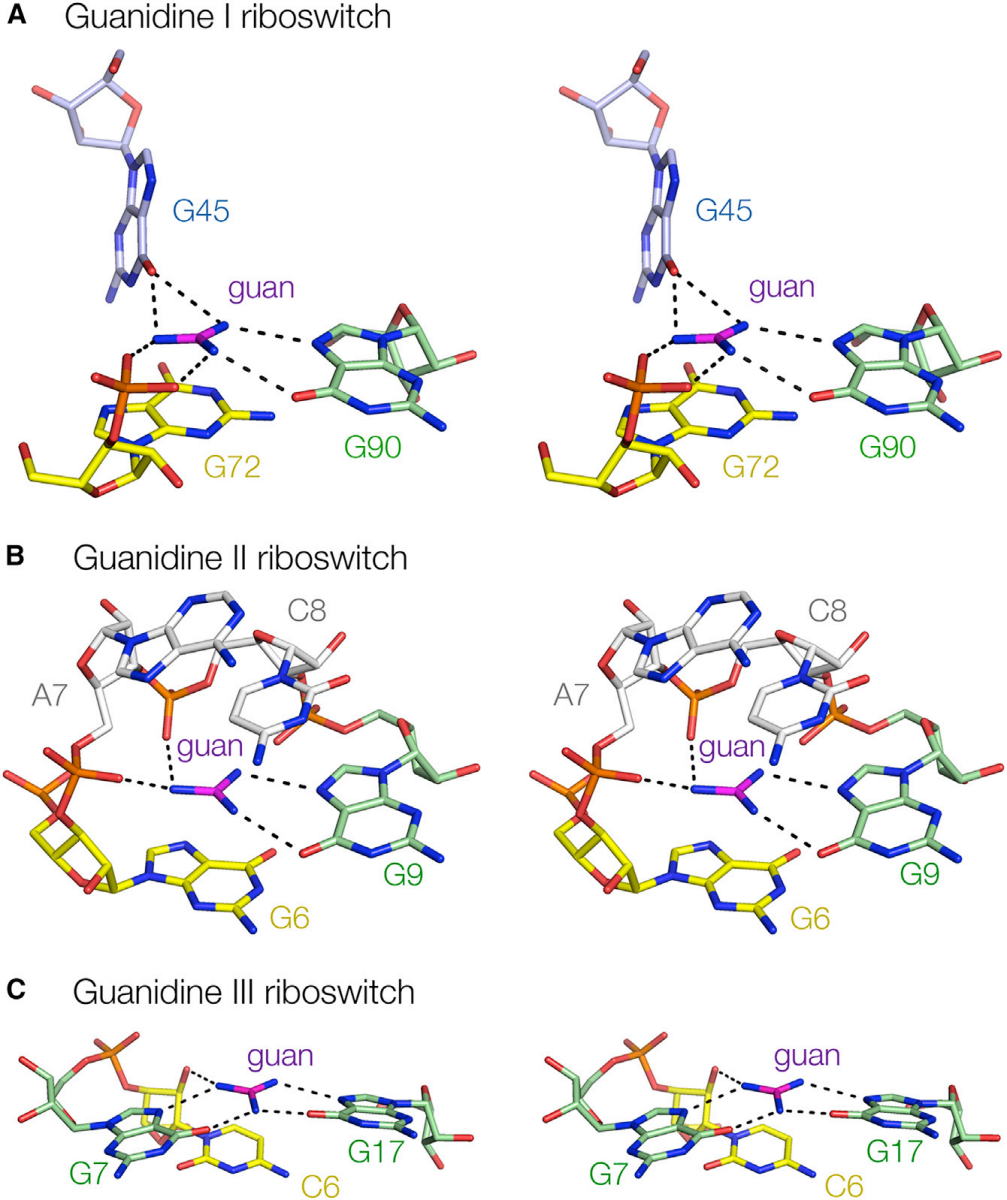
Comparison of the Guanidine Binding Sites of the Three Classes of Guanidine Riboswitch Structures The nucleotides have been colored to reflect their function in ligand binding. Guanine bases accepting hydrogen bonds from guanidine at O6 and N7 on the Hoogsteen edge are green (all classes), guanine bases making hydrogen bonds to O6 alone (A guanidine I riboswitch) are blue, nucleotides only making backbone contacts (B guanidine II riboswitch) are gray, and nucleotides making π-cation interaction with the guanidinium cations (all classes) are yellow. The image of the guanidine I riboswitch was prepared using PDB: 5T83 (Reiss et al., 2017), and that of the guanidine II riboswitch (P1 stem-loop) was prepared using PDB: 5NDI (Huang et al., 2017). The guanidine III riboswitch (C) is the present work.

The guanidine binding sites of the three riboswitches exhibit a number of common features as well as some differences (Figure 7). All involve guanine nucleobases, and in each case two guanidine nitrogen atoms are bonded to O6 and N7 on the Hoogsteen edge. In the guanidine III riboswitch, this occurs twice in the binding site. This is also a common feature in RNA-protein interactions, where the guanidino group of an arginine side chain frequently interacts with O6 and N7 of guanine, e.g., in the Tat-TAR interaction (Puglisi et al., 1993) and in zinc-finger proteins such as Zif268 (Pavletich and Pabo, 1991). Contact with the RNA backbone is universal, yet the three riboswitches do this in different ways. Guanidine is hydrogen bonded to bridging or non-bridging phosphate oxygen atoms in both the guanidine I and II riboswitches, while a 2′-hydroxyl group participates as the sole backbone contact to the ligand in the guanidine III riboswitch. Lastly, for each riboswitch it is observed that the guanidinium cation is stacked on a nucleobase at a distance of 3.5 Å, consistent with π-cation interactions (Gallivan and Dougherty, 1999, Wintjens et al., 2000). In both the guanidine I and II riboswitches the nucleobase is guanine, whereas in the guanidine III riboswitch it is a cytosine. The calculated energy of interaction between methylammonium and benzene is −5.5 kcal mol^−1^ in water (Gallivan and Dougherty, 2000). The interaction would be anticipated to be stronger for the purine, and the selection of a pyrimidine in the guanidine III riboswitch may reflect an evolutionary weakening of the interaction in order to tune the riboswitch to the required physiological response range.

Clearly specificity of binding is as important to riboswitch function as is affinity. All three guanidine riboswitches are highly selective, in each case excluding the rather similar metabolite urea (Nelson et al., 2017, Sherlock and Breaker, 2017, Sherlock et al., 2017). The guanidine I riboswitch forms six hydrogen bonds to the guanidine ligand, and is completely selective for that ligand alone (Battaglia et al., 2017, Reiss et al., 2017). By contrast, the guanidine II and III riboswitch RNAs make hydrogen bonds with four or five guanidino protons, and each has a small opening at the side that will accommodate a small side chain. Like the guanidine II riboswitch (Huang et al., 2017), we present here structures with methyl-, ethyl-, and aminoguanidine bound, showing that the additional alkyl and amino groups are tolerated within the structure. These occupy the position taken by an ordered water molecule in the structure with bound guanidine. We also show that agmatine may bind to the guanidine III riboswitch, but in a different manner that involves a rotation of the guanidino nucleus.

Finally, although all three guanidine riboswitches function as ON switches, they do not do so in the same way. The guanidine I riboswitch has been demonstrated to act as a transcriptional regulator (Nelson et al., 2017), preventing a transcriptional terminator stem-loop from forming when folded with guanidine ligand bound (Battaglia et al., 2017, Reiss et al., 2017). The functional mechanisms of the guanidine II and III riboswitches are less well established, but in both cases the proximity to ribosome binding sites suggests that ligand-induced folding exposes the site so that translation can be initiated. In each case the net effect is that, when the cellular concentration of guanidine rises into the micromolar concentration range, it binds to and folds each riboswitch structure resulting in the expression of enzymes and efflux pumps that deal with this highly toxic compound. Yet comparison of these three riboswitches shows how evolution works on RNA structure to find three quite different folds that achieve the same result.

## Significance

**The guanidine III riboswitch is the third member of the class of guanidine-responsive riboswitches. X-ray crystallography shows that the RNA folds into an H-type pseudoknot, in which a connecting strand locates in the major groove of one duplex to form a regular triple helix. This continues into the central core of conserved nucleotides that form a novel left-handed helical ramp of inter-nucleotide interactions, generating the guanidinium cation binding site. The guanidine ligand is hydrogen bonded to O6 and N7 of two guanine nucleobases, and to the 2′-OH of a cytidine, the nucleobase of which interacts with the guanidinium in a π-cation interaction. The nature of the binding pocket shows why urea is excluded, but there is a side opening that can accommodate small side chains, and we have determined structures with bound methylguanidine, aminoguanidine, ethylguanidine, and agmatine. Comparison with earlier probing data suggest that the formation of the triple helix is stabilized by guanidine binding and this exposes the adjacent ribosome binding site to permit initiation of gene translation.**

## STAR★Methods

### Key Resources Table

**Table:** 

REAGENT or RESOURCE	SOURCE	IDENTIFIER
**Chemicals, Peptides, and Recombinant Proteins**		

Guanidine hydrochloride	Sigma-Aldrich	50993
Aminoguanidine hydrochloride	Sigma-Aldrich	396494
Methylguanidine hydrochloride	Sigma-Aldrich	222402
Agmatine sulfate	Sigma-Aldrich	A7127
1-Ethylguanidine sulfate	Sigma-Aldrich	275557
5-bromocytidine	ChemGenes	ANP-5648
Triethylamine trihydrofluoride	Sigma-Aldrich	344648

**Deposited Data**		

The structure of the thermobifida fusca guanidine III riboswitch with guanidine in space group P3_1_21.	This paper	5NWQ
The structure of the thermobifida fusca guanidine III riboswitch with guanidine in space group P3_2_12.	This paper	5NZ6
The structure of the thermobifida fusca guanidine III riboswitch in space group P2_1_2_1_2_1_	This paper	5NZD
The structure of the thermobifida fusca guanidine III riboswitch with aminoguanidine.	This paper	5NY8
The structure of the thermobifida fusca guanidine III riboswitch with methylguanidine.	This paper	5NZ3
The structure of the thermobifida fusca guanidine III riboswitch with 1-Ethylguanidine.	This paper	5O62
The structure of the thermobifida fusca guanidine III riboswitch with agmatine.	This paper	5O69

**Oligonucleotides**		

Thermobifida fusca guanidine III riboswitch RNA sequence: C(BrC)GGACGAGGUGCGCCGUACCCGGUCAGGACAAGACGG(BrC)GC	This paper	N/A

**Software and Algorithms**		

Phenix	(Adams et al., 2010)	http://phenix-online.org
COOT	(Emsley et al., 2010)	https://www2.mrc-lmb.cam.ac.uk/personal/pemsley/coot/
XIA2 version 0.5.270	(Winter, 2010)	https://xia2.github.io

### Contact for Reagent and Resources Sharing

Professor David M. J. Lilley FRS : d.m.j.lilley@dundee.ac.uk

### Experimental Model and Subject Details

All RNA used in our crystallographic studies was made by chemical synthesis. No animals or cell lines have been used in this work.

### Method Details

#### RNA Synthesis

RNA oligonucleotides were synthesized using ***t***-BDMS phosphoramidite chemistry (Beaucage and Caruthers, 1981) as described in Wilson et al. (Wilson et al., 2001), implemented on an Applied Biosystems 394DNA/RNA synthesizer. Oligonucleotides containing 5-bromocytidine (ChemGenes) were deprotected in a 25% ethanol/ammonia solution for 36 h at 20°C. All oligoribonucleotides were redissolved in 100 μL of anhydrous DMSO and 125 μl triethylamine trihydrofluoride (Aldrich) to remove *t*-BDMS groups, and agitated at 65°C in the dark for 2.5 h. After cooling on ice for 10 min, the RNA was precipitated with 1 mL of butanol, washed twice with 70 % ethanol and suspended in double-distilled water.

RNA was further purified by gel electrophoresis in polyacrylamide under denaturing conditions in the presence of 7 M urea. The full-length RNA product was visualized by UV shadowing. The band was excised and electroeluted using an Elutrap Electroelution System (GE Healthcare) into 45 mM Tris-borate (pH 8.5), 5 mM EDTA buffer for 8 h. at 200 V at 4°C. The RNA was precipitated with ethanol, washed once with 70 % ethanol and suspended in double-distilled water. The RNA sequence used for crystallization was (5' to 3') :

C(BrC)GGACGAGGUGCGCCGUACCCGGUCAGGACAAGACGG(BrC)GC where BrC is 5-bromocytosine.

#### Chemicals and Reagents

Guanidine, methylguanidine and aminoguanidine were used as hydrochlorides, while ethylguanidine and agmatine were used as sulfates. All were purchased as the highest available grade from Sigma-Aldrich.

#### Crystallization, Structure Determination, and Refinement

A solution of 0.6 mM RNA in 5 mM HEPES (pH 7.6), 100 mM KCl was heated to 95°C for 1 min. The solution was slowly cooled to 20°C and MgCl_2_ was added to a final concentration of 2 mM. Guanidine, methylguanidine and aminoguanidine were added to a final concentration of 10 mM and cocrystallized with the RNA. 10 mM ethylguanidine and 100 mM agmatine were soaked into crystals of *T. fusca* ligand-free RNA using the conditions indicated in Table S1.

Diffraction data were collected on beamline I03 of Diamond Light Source (Harwell, UK). Most of the data were collected under rapid access route, proposal number MX17492. The data with bound ethylguanidine and agmatine were collected under proposal number MX14980.

Data were processed by XIA2 version 0.5.270 (Winter, 2010). The resolution cutoff for the data was determined by examining by CC1/2 and density map as described previously (Karplus and Diederichs, 2012). Initial phase information for 5NWQ, 5NZD, 5NY8, 5NZ3, 5O63 and 5O69 were acquired from the SAD data by locating the bromine atoms with Autosol in the PHENIX suite (Adams et al., 2010). Structure 5NZ6 was determined by molecular replacement using the 5NWQ as the initial model. Models were adjusted manually using Coot (Emsley et al., 2010) and subjected to several rounds of adjustment and optimization using Coot and phenix.refine. Model geometry and the fit to electron density maps were monitored with MOLPROBITY (Chen et al., 2010) and the validation tools in Coot. The unbiased electron density map was generated through Br-SAD phasing and density modification by PHENIX AutoSol.

### Data and Software Availability

All software were reported in Method Details and indicated in the Key Resources Table.

The accession numbers for the coordinates and structure factors of all structures in this paper have been deposited in the PDB indicated in the Key Resources Table.

## Author Contributions

L.H., J.W., and D.M.J.L. planned the experiments. L.H. and J.W. performed crystallography. L.H., T.W., and D.M.J.L. analyzed data and wrote the paper.

## Supplemental Information

Document S1. Figures S1–S5 and Tables S1–S4

Document S2. Article plus Supplemental Information
