# Supplementary material for: Structure of the Guanidine III Riboswitch
Source: Cell Chem Biol. 2017 Nov 16;24(11):1407–1415.e2. doi: 10.1016/j.chembiol.2017.08.021 (PMC5696562; doi:10.1016/j.chembiol.2017.08.021)
Supplement: Document S1. Figures S1–S5 and Tables S1–S4 [file mmc1.pdf]

Cell Chemical Biology, Volume *24*

Supplemental Information

Structure of the Guanidine III Riboswitch

Lin Huang, Jia Wang, Timothy J. Wilson, and David M.J. Lilley

The structure of the guanidine-III riboswitch

Lin Huang, Jia Wang, Timothy J. Wilson and David M. J. Lilley

SUPPLEMENTARY INFORMATION

Comprising five supplementary figures and four supplementary tables.

SUPPLEMENTARY FIGURES

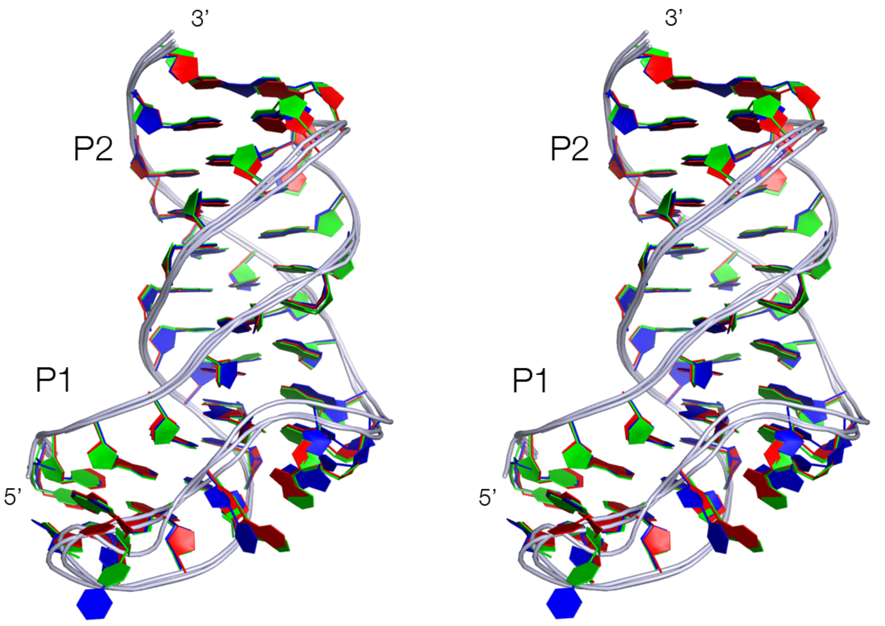

Figure S1. Superposition of the structures of the guanidine III riboswitch determined in three different space groups. The crystallographic symmetries are $P3_121$ (green), $P3_212$ (blue) and $P2_12_12_1$ (red). The core of the riboswitch is almost perfectly superimposed. The largest deviation occurs in the third strand of the P1 helix. A Parallel-eye stereoscopic pair is shown. Related to Figure 1.

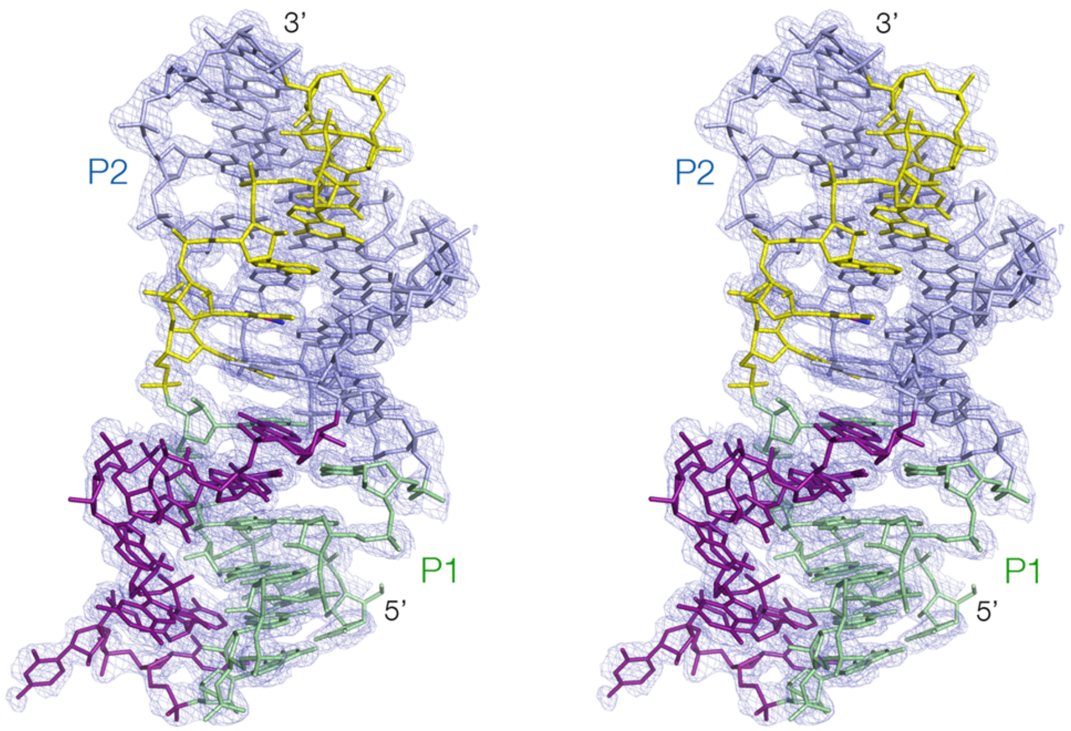

Figure S2. The overall conformation of the guanidine III riboswitch, showing the experimental phasing map contoured at 1.2 σ. The view is similar to that shown in Figure 1C. A parallel-eye stereoscopic pair is shown. Related to Figure 1.

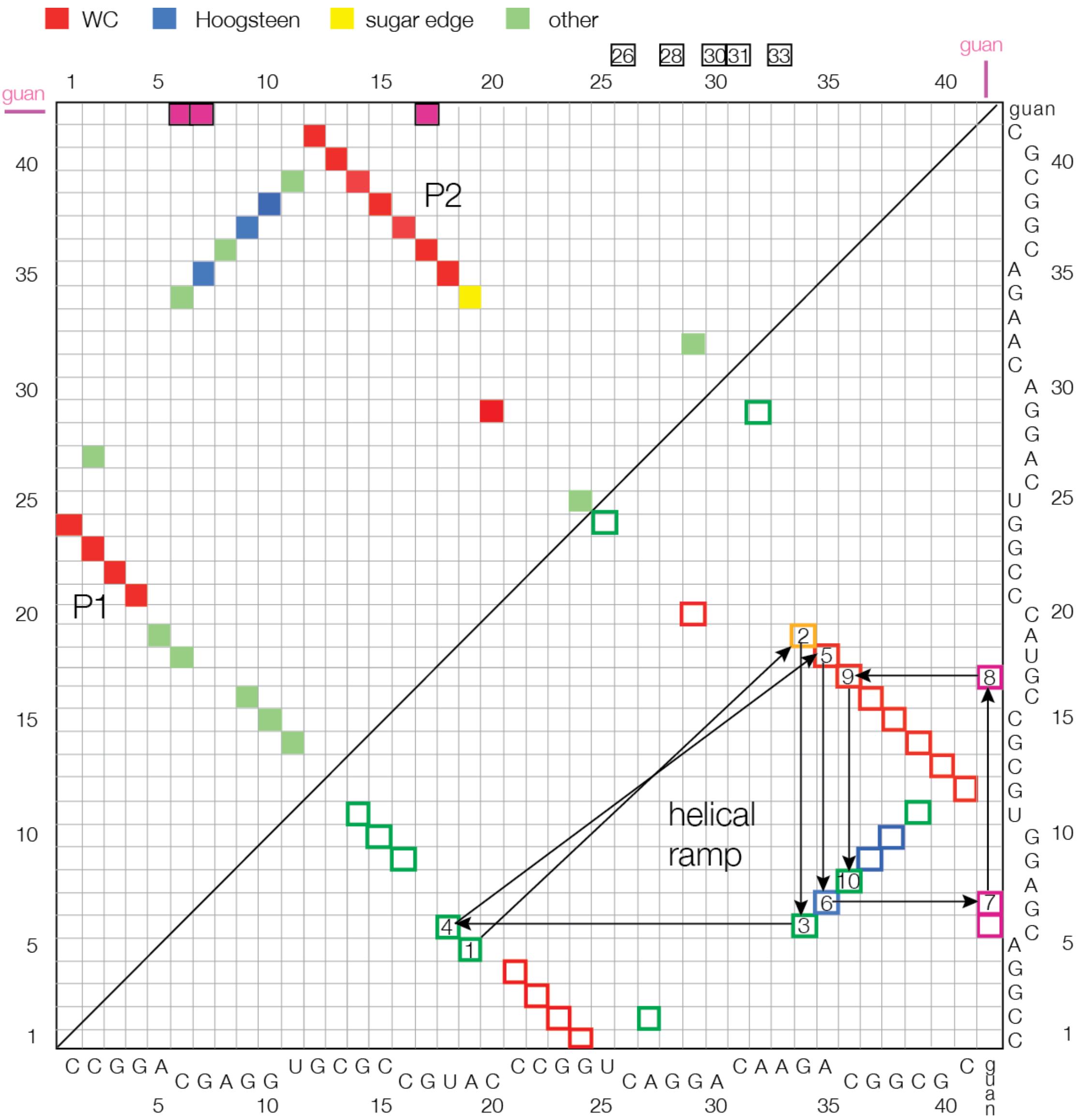

Figure S3. A two-dimensional plot of inter-nucleotide interactions. Each square represents an interaction between two nucleotides, colored according to the nature of the interaction; the key is written at the top. guan = guanidine. Boxed nucleotides (top) are not involved in hydrogen bonding to other nucleotides. The plot is symmetrical about the diagonal. In the lower half the sequential interactions of the helical ramp are indicated by the arrows. The internucleotide contacts plotted here are listed sequentially in Table S3. Related to Figure 1.

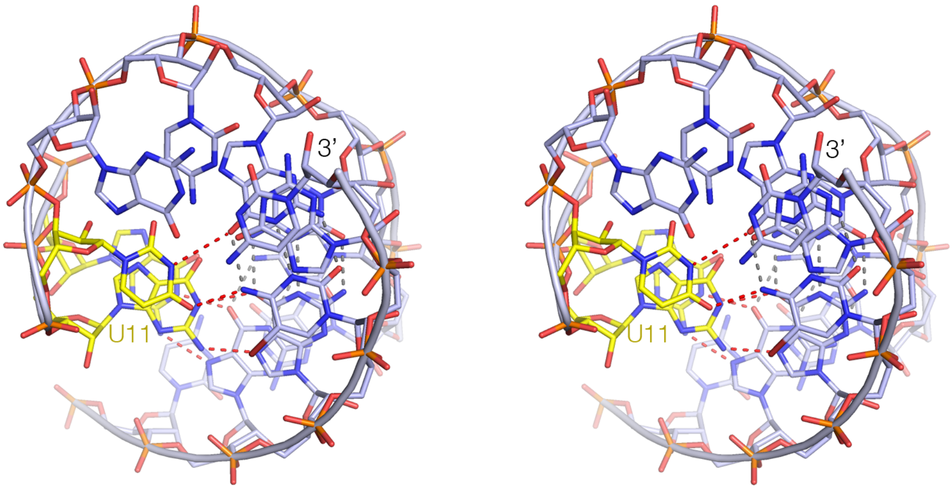

Figure S4. The P2 triple helix viewed down its axis. A parallel-eye stereoscopic pair of the axial view, showing the third strand (yellow) enclosed deep in the major groove of P2. Hydrogen bonds between the third strand and the P2 duplex are colored red. Related to Figure 3.

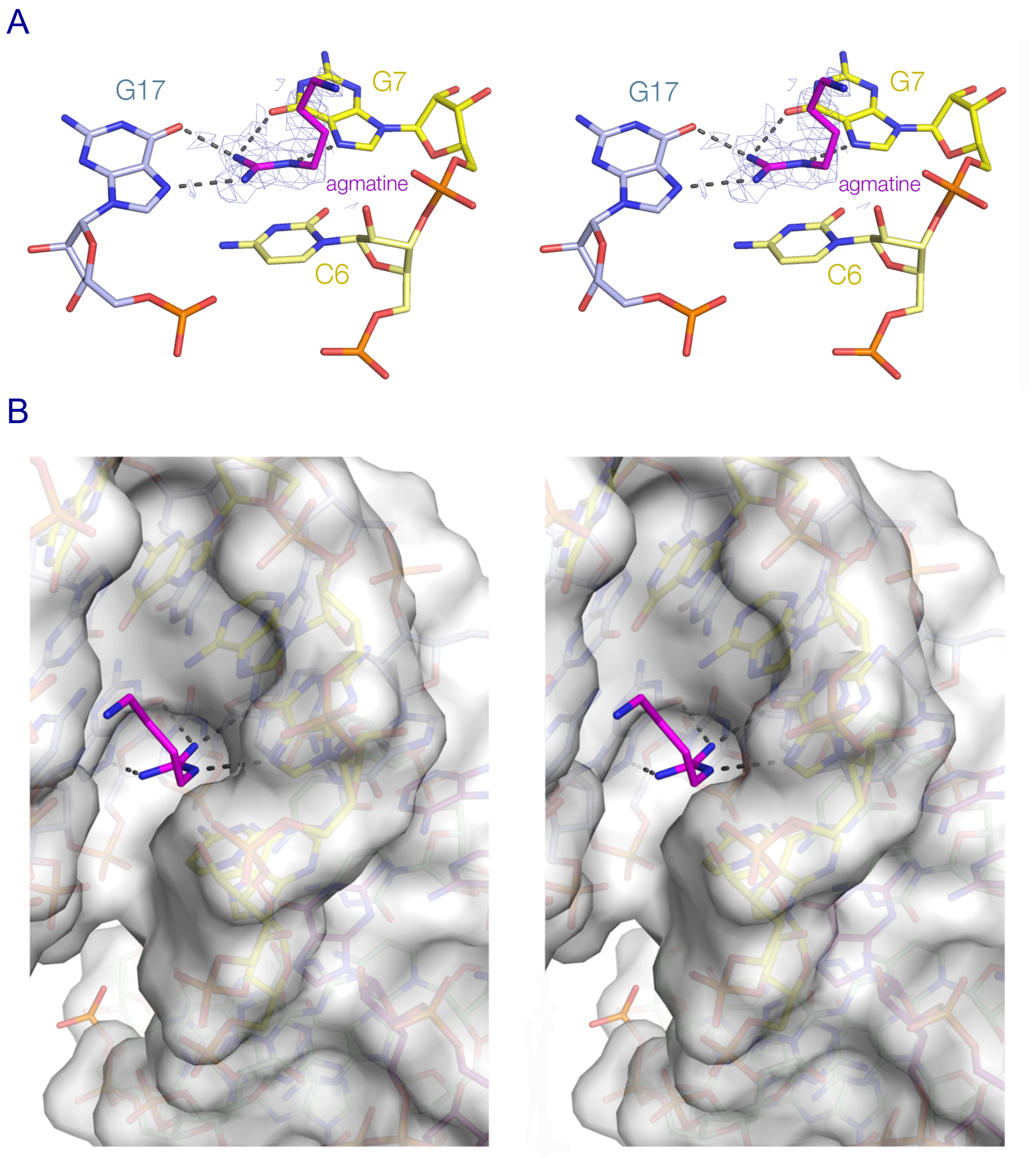

Figure S5. The structure of agmatine bound into the binding pocket of the guanidine III riboswitch. Parallel-eye stereoscopic pairs are shown.

A. The experimental phasing map of agmatine bound to the riboswitch contoured at 1.2 σ. Note that is contrast to the other guanidine variants, the butylamine sidechain is attached to the nitrogen atom bound to G7 N7 rather than G17 N7.

B. View into the side opening of the binding pocket, showing how the butylamine sidechain of the agmatine is directed along the groove. Related to Figure 6.

SUPPLEMENTARY TABLES

Table S1.

PDB	ligands	mother liquor	r (K)	Resol (Å)	space group
5NWQ	cocrystallized 10 mM guanidine	0.1 M sodium chloride, 0.02 M magnesium chloride hexahydrate, 0.05 M Bis-tris (pH 7.0), 30% v/v MPD	280	1.91	$P3_121$
5NZ6	cocrystallized 10 mM guanidine	60% v/v tacsimate (pH 7.0)	293	2.94	$P3_212$
5NZD	ligand-free (acetate)	0.1 M magnesium acetate, 0.05 M MES (pH 5.6), 20% v/v MPD	293	2.01	$P2_12_12_1$ *
5NY8	cocrystallized 10 mM aminoguanidine	0.1 M sodium chloride, 0.02 M magnesium chloride hexahydrate, 0.05 M Bis-tris (pH 7.0), 30% v/v MPD	293	2.04	$P3_121$
5NZ3	cocrystallized 10 mM methylguanidine	0.1 M magnesium acetate, 0.05 M MES pH 5.6, 20% v/v MPD	293	2.06	$P3_121$
5O62	soaking with 10 mM 1-ethylguanidine	0.1 M magnesium acetate, 0.05 M MES (pH 5.6), 20% v/v MPD	293	2.12	$P3_121$
5O69	soaking with 100 mM agmatine	0.1 M magnesium acetate, 0.05 M MES (pH 5.6), 20% v/v MPD	293	2.32	$P3_121$

Table S1. Summary of the ligands and crystallization conditions used in these experiments, and the crystals obtained. Related to Figure 1.
* crystallization in the absence of added ligand gave crystals in multiple space groups. The structure was determined in $P2_12_12_1$.

Table S2.

ligands	guanidine	guanidine	ligand-free	amino guanidine	methyl guanidine	1-ethyl guanidine	agmatine
PDB	5NWQ	5NZ6	5NZD	5NY8	5NZ3	5O62	5O69
**Data collection**							
Space group	$P3_121$	$P3_212$	$P2_12_12_1$	$P3_121$	$P3_121$	$P3_121$	$P3_121$
Cell dimensions							
a, *b*, *c* (Å)	83.6, 83.6, 66.6	83.6, 83.6, 98.8	49.5, 64.6, 84.3	83.4, 83.4, 66.3	83.5, 83.5, 66.1	84.0, 84.0, 67.4	83.9, 83.9, 67.2
a, *b*, *g* (°)	90 90 120	90 90 120	90 90 90	90 90 120	90 90 120	90 90 120	90 90 120
	SAD-Br	MR	SAD-Br	SAD-Br	SAD-Br	SAD-Br	SAD-Br
	Peak		*Peak*	*Peak*	*Peak*	*Peak*	*Peak*
Wavelength	0.9202	0.9202	0.9202	0.9202	0.9202	0.9197	0.9197
Resolution (Å)	41.80 – 1.91 (1.94 – 1.91)	49.41 – 2.94 (2.99 – 2.94)	42.15 - 2.01 (2.04 – 2.01)	25.96 - 2.04 (2.08 – 2.04)	48.77 – 2.06 (2.09 - 2.06)	36.38 – 2.12 (2.20 -2.12)	49.34 – 2.32 (2.36-2.32)
R_{merge}	0.042 (1.100	0.101 (1.658)	0.049 (1.282)	0.049 (1.023)	0.106 (2.233)	0.120 (2.877)	0.181 (3.091)
I / s*I*	16.5 (1.6)	18.2 (1.7)	11.2 (1.4)	16.7 (1.7)	11.2 (1.2)	16 (1.3)	13.2(1.4)
CC (1/2)	1.00 (0.57)	0.97 (0.96)	0.99 (0.82)	1.00 (0.54)	1.00 (0.72)	1.00 (0.50)	1.00 (0.589)
Completeness (%)	100 (100)	100 (100)	99.8 (96.1)	99.6 (100)	100 (95.7)	100 (100)	100 (98.1)
Redundancy	5.3 (5.4)	21.6 (21.1)	5.1 (5.2)	5.6 (5.7)	15.1 (14.5)	21.3 (21.9)	29.2 (30.3)
**Refinement**							
Resolution (Å)	36.2 – 1.91 (1.98 – 1.91)	32.94 – 2.94 (3.05 – 2.94)	42.15- 2.01 (2.08 – 2.01)	25.96- 2.04 (2.11 – 2.04)	48.77 – 2.06 (2.13 – 2.06)	36.38 - 2.12 (2.20 - 2.12)	36.32 – 2.32 (2.40 - 2.32)
No. reflections	21226 (2097)	8571 (820)	18298 (1765)	17219 (1688)	16345 (1589)	15972 (1594)	12159 (1187)
R_{work} / R_{free}	0.205 / 0.225	0.194 / 0.215	0.227 / 0.250	0.195 / 0.236	0.231 / 0.287	0.215 / 0.264	0.247 / 0.273
No. atoms							
Macromolecules	1722	838	1682	1722	1682	1510	1593
ligands	64	48	107	53	102	95	98
B-factors							
Macromolecules	76.5	111.9	62.00	90.54	71.42	62.56	73.86
ligands	76.1	138.7	62.28	59.67	60.48	61.86	86.46
Solvent	55.1		60.96	47.89	55.04	49.67	55.46
R.m.s. deviations							
Bond lengths (Å)	0.004	0.009	0.005	0.008	0.008	0.006	0.004
Bond angles (°)	0.76	1.62	0.92	1.28	1.40	1.08	0.82

*Values in parentheses are for highest-resolution shell.

Table S2. Details of data collection and refinement statistics for the crystallographic data as deposited with the PDB. Related to Figure 1.

Table S3.

first nt	WC	non WC	notes
C1	G24		P1
C2	G23		P1
O2'		A27 N1	
G3	C22		P1
G4	C21		P1
A5 N6 N1		A19 N7 N6	
C6 N4		U18 O4	
O2'		guanidine	
O2 N3		G34 N1 N2	~wobble
G7 N1 O6		A35 N7 N6	*cis* Hoogsteen
O6 N7		guanidine	
A8 N1		C36 N4	
G9 O6		C16 N4	
N1 N2		G37 O6 N7	Hoogsteen
G10 N1 N2		G38 O6 N7	Hoogsteen
O6		C15 N4	
U11 N3		G14 O6	Tight turn
O4		C39 N4	
G12	C41		P2
C13	G40		P2
G14	C39		P2
		U11	
C15	G38		P2
		G10	
C16	G37		P2
		G9	
G17	C36		P2
O6 N7		guanidine	
U18	A35		P2
		C6	
A19		A5	
N1 N6		G34 O2' N3	sugar edge
C20	G29		P1
C21	G4		P1
C22	G3		P1
G23	C2		P1
G24	C1		P1
N2		U25 O4	
U25		G24	turn, loop

C26			no Hbo contacts
A27		C2	
G28			no Hbo contacts; stacked on G30
G29	C20		P1 extension; backbone extended
N2 N3		A32 N1 N6	
A30			no Hbo contacts; stacked between G28, A31
C31			no Hbo contacts; stacked between A30, A32
A32		G29	stacked
A33			no Hbo contacts; stacked between A32, A34
G34		C6	
A35	U18		P2
		G7	P2 third strand
C36	U17		P2
		A8	P2 third strand
G37	C16		P2
		G9	P2 third strand
G38	C15		P2
		G10	P2 third strand
C39	G14		P2
		U11	P2 third strand
G40	C13		P2
C41	G12		P2

Table S3. List of internucleotide contacts in the guanindine III riboswitch structure. Contacts to the guanidine ligand are highlighted yellow, and P1 and P2 helices are highlighted grey. *Hbo* = hydrogen bond. The internucleotide contacts listed here are plotted in Figure S2. Related to Figures 1,2, 3 and 4.

Table S4.

guanidine riboswitch	I	II	III
mode of control	transcription	translation	translation
overall structural fold	2 helices	2 stem-loops	pseudoknot
	packed laterally	loop-loop	triple helix
ligands bound	1	2	1
ligand contacts :			
guanidine protons donated	6	4	5
G-Hoogsteen	1	1	2
π-cation	G	G	C
backbone	NBO, BO	2xNBO	O2'
urea discrimination	+	+	+
toleration of extra atoms	-	+	+

Table S4. Comparison of the structural properties and ligand binding sites of the guanidine I, II and III riboswitches. Related to Figure 7.

NBO : Non-bridging phosphate oxygen atoms; BO : Bridging phosphate oxygen atoms
